# Robust EMG sensing system based on data fusion for myoelectric control of a robotic arm

**DOI:** 10.1186/1475-925X-8-5

**Published:** 2009-02-25

**Authors:** Natalia M López, Fernando di Sciascio, Carlos M Soria, Max E Valentinuzzi

**Affiliations:** 1 Gabinete de Tecnología Médica, Facultad Ingeniería, Universidad Nacional de San Juan, San Juan, Argentina; 2 Instituto de Automática, Facultad Ingeniería, Universidad Nacional de San Juan, San Juan, Argentina; 3 Instituto Superior de Investigaciones Biológicas, CONICET, Tucumán, Argentina

## Abstract

**Background:**

Myoelectric control of a robotic manipulator may be disturbed by failures due to disconnected electrodes, interface impedance changes caused by movements, problems in the recording channel and other various noise sources. To correct these problems, this paper presents two fusing techniques, Variance Weighted Average (VWA) and Decentralized Kalman Filter (DKF), both based on the myoelectric signal variance as selecting criterion.

**Methods:**

Tested in five volunteers, a redundant arrangement was obtained with two pairs of electrodes for each recording channel. The myoelectric signals were electronically amplified, filtered and digitalized, while the processing, fusion algorithms and control were implemented in a personal computer under MATLAB^® ^environment and in a Digital Signal Processor (DSP). The experiments used an industrial robotic manipulator BOSCH SR-800, type SCARA, with four degrees of freedom; however, only the first joint was used to move the end effector to a desired position, the latter obtained as proportional to the EMG amplitude.

**Results:**

Several trials, including disconnecting and reconnecting one electrode and disturbing the signal with synthetic noise, were performed to test the fusion techniques. The results given by VWA and DKF were transformed into joint coordinates and used as command signals to the robotic arm. Even though the resultant signal was not exact, the failure was ignored and the joint reference signal never exceeded the workspace limits.

**Conclusion:**

The fault robustness and safety characteristics of a myoelectric controlled manipulator system were substantially improved. The proposed scheme prevents potential risks for the operator, the equipment and the environment. Both algorithms showed efficient behavior. This outline could be applied to myoelectric control of prosthesis, or assistive manipulators to better assure the system functionality when electrode faults or noisy environment are present.

## Background

Teleoperation of robotic devices for rehabilitation and assistive tasks has increased in later years due, in part, to the introduction of simple interfaces with the ability of discerning the operator's intent [1]. Surface electromyography (EMG) represents an efficient signal for control purposes. Furthermore, the operator's movement is not perturbed by the surface electrodes, allowing an easier adaptation to assistive devices.

The high gain amplification required due to the low level of EMG signals makes myoelectric control rather sensitive to amplitude changes. Such variations can lead to difficulties because the controller might receive incompatible values with the robot specifications, i.e., the mechanical articulations may be subjected to displacements or velocities larger than the recommended ranges, which, in turn, lead to the activation of the robot's protection system.

Typical failures in the case of bioelectric potentials are broken electrode connections or sudden changes in the electrode-electrolyte interface due, for example, to the operator's movements or poor contact, which lead to direct input of noise into the control system. Besides, in the situation herein described, some noise is added by the robot power supplies. If all of the above-mentioned potential failures are not properly considered, they may result in damages to the system, the user or even to third parties.

Robots used, say, in service and rehabilitation of the aged or people with disabilities, execute tasks in cooperation with other humans, so that safety considerations are needed. In order to avoid any possible operator's injury, and/or the activation of the robot safety system, various strategies have been proposed in the literature. The method proposed by Kulic [2] is based on the trajectory planning with the inclusion of the operator's position; in Fukuda *et al *[3], instead, entropy appears as good risk indicator of an incorrect or ambiguous command, that is, when the entropy goes beyond a specified threshold level the robot motors stop. In another approach, Fleischer [4] introduces a soft manipulator with a flexible joint composed of an electro-rheological fluid and a torque controller considering human pain tolerance. To assure safety of an exoskeleton for the knee joint support, in [5] all sensor data are range-checked and clipped to sensitive boundaries by software, besides other mechanical considerations.

All safety considerations mentioned above prevent human risks by different methods, like stopping the motors or including the operator's position data. The aim of this work is to guarantee the correct and continuous functioning of the system, even in case of failures. For this purpose, two data fusion strategies are proposed, Variance Weighted Average (VWA) and Decentralized Kalman Filter (DKF) [6], by means of an arrangement of redundant potentials, that is, combining the EMG signals from two or more acquisition channels in such a way that after the fusion stage, the algorithms provide a more reliable signal to be applied to the control system.

Data fusion techniques are frequently implemented in robotic control, where the information is redundant and/or of diverse nature [7,8]. When the data sensors are similar, fusion is applied over the signals, but when the data sensors are of different nature, fusion takes place on the control signals [8]. In this application, the interested variables are measured by two or more pairs of electrodes to obtain more information than from a single channel. The sensors (electrodes) differ only in their location and not in their characteristics, and their signals are fused to reduce the sensitivity of the control system relative to electrode failures, so increasing the overall robustness.

The paper is organized as follows: Methods-A presents the system and processing overview. The methodology of acquisition and robot control is discussed in Methods-B and the algorithms for data fusion in Methods-C. In Results, Section A, the two fusion techniques are applied to EMG signals in the presence of failures while a comparative performance under simulated noise conditions is given in Section B. Finally, in the Discussion, the merits and limitations of these algorithms are discussed.

## Methods

### Equipment and processing

**Figure 1:**
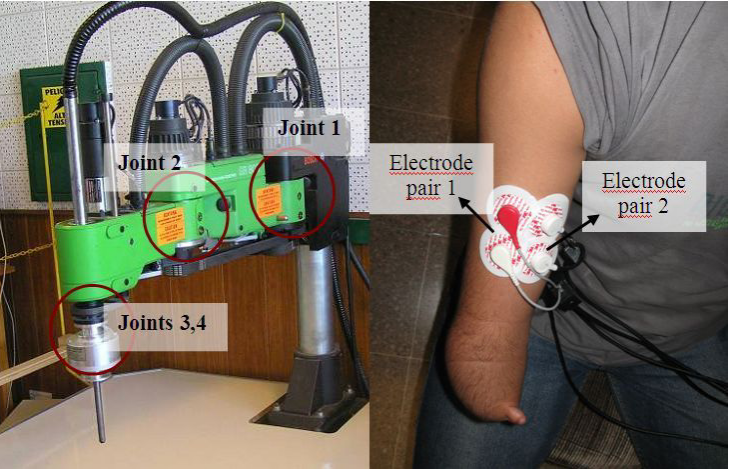
**Experimental set-up used for data acquisition**. (Left) BOSCH SR-800. The circles indicate the joints of the robot. Joint 1 is the used in the experiments; (Right) Amputee volunteer with an arrangement of electrodes on his stump.

All the experiments were performed on an industrial robotic manipulator BOSCH SR-800, type SCARA (*Selective Compliance Assembly Robot Arm*), with four degrees of freedom and a horizontal reach of 800 mm (Fig. 1). To demonstrate the validity of the algorithms herein proposed, only one degree of freedom was used, in this case the first joint (shoulder). For parameter adjustment, the sensor data acquisition and fusion algorithms were carried out off-line in MATLAB-SIMULINK^® ^(The Mathworks, Natick, MA), while the real time control algorithms were finally implemented in a Freescale^® ^kit DSP56F801 programmed in C++^®^.

Following the recommendations of SENIAM protocol [9], bipolar EMG's were recorded with a pair of Ag/AgCl electrodes (*3M RedDot*) placed 20 mm apart. The longitudinal axis of this pair was aligned, when possible (in amputees such positioning may not be realized), along the muscle fibers. An array of two or more identical pairs of electrodes was placed in the volunteer's arm (Fig. 1).

Electronic amplification, optical isolation, and filtering are implemented by a custom-made front-end signal conditioning circuit with the following characteristics:

Amplification stage (AD620, Analog Devices^®^): Input impedance: 10 MΩ; Gain: 1000; Common Mode Rejection Ratio (CMRR): 120 dB.

Filter: 6^th ^order Band-Pass Filter with cuttoff frequencies 10–500 Hz and Butterworth Coefficients

Thereafter, the analogue EMG signals were digitalized at a sampling rate of 1 KHz with an A/D 6024-E board (National Instruments^®^) and processed according to Table 1 under MATLAB^® ^as preliminary stage. In the end, the real time system was implemented in a DSP kit (DSP56F801, FREESCALE^®^) [10].

**Table 1:** Sequence of operators applied to EMG signal.

Filtering with a 6^th ^order Butterworth Bandpass filter (10 Hz–500 Hz).	*emg*_*filt*_(*k*) = *filter *(*emg*_*raw*_(*k*))
Normalization with respect to MVC	$emg_{norm}(k)=\frac{emg_{filt}(k)}{MVC}$

Full-wave rectification	*emg*_*rect*_(*k*) = *abs *(*emg*_*norm*_(*k*))

Background noise removing Symmetric Dead Zone *BNT = Background Noise Threshold*	*emg*(*k*) = DeadZone(*emg*_*norm*_(*k*)) = if *abs *(*emg*_*norm*_(*k*)) ≥ *BNT; emg*(*k*) = *emg*_*norm*_(*k*) *otherwise emg(k) = 0*

Even when the relationship between EMG amplitude and muscular force is controversial, some features are accepted as estimators in myoelectric control, especially in amputees, due to the impractical measure of the muscle force [11]. Several factors, like electrode location, interelectrode distance, subcutaneous fat layer thickness, make it impossible to consider a generalization of the EMG-force relation in different subjects and experimental sessions [12]. Nevertheless, in the initial setting stage, the system records the maximum voluntary contraction (MVC) and the background noise applying the same procedure described in Table 1. Hence, these values were calculated over the rectified and smoothed signals. Afterwards, with this information, the system executes an adaptive routine for the current environmental conditions and for the specific characteristics of each volunteer. Furthermore, the relation between the muscle force and the command for the robot control was obtained from pairs of agonist-antagonist muscles. This is because the operator was trained based on the functional muscle group, which reduces the influence of each individual muscle.

**Figure 2:**
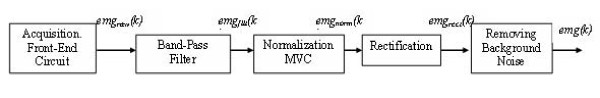
**Processing scheme of the EMG signal**. Full description can be found in Table 1.

The EMG often shows slow variations due to movement artifacts and instability of the electrode-skin interface, therefore, a sixth order Butterworth band-pass filter was implemented. It removed low frequency components (below 10 Hz) and limited the EMG bandwidth below 500 Hz to prevent high frequency noise amplification. Thereafter, the signal was full-wave rectified and normalized with respect to the MVC. Finally, the background noise was removed by applying a symmetric *dead-zone operator*; this is done to eliminate the arm drift caused by the cumulative effects of the background noise after full-wave rectification. Figure 2 and Table 1 summarize the processing steps of the raw EMG.

Regarding the rejection of the noise generated by the power source, the use of notch filters is not recommended in EMG applications because they introduce phase rotation and remove a frequency band, precisely where this signal shows an important power density [12]. The high CMRR of the differential amplifier (120 dB) improves noise rejection.

EMG is presented as a time sequence, which must be mapped to a smaller dimension vector by the computation of several features leading to a muscle force estimator and input to the classifier. A wide spectrum of features can be found in the literature, computed either in the time or frequency domain, or both, as can be seen in [13] and the references therein cited. Time domain features are widely used due to computational simplicity and real-time control possibilities. For choosing the most adequate, the statistical set proposed by [14] was evaluated in terms of computational cost and repeatability.

Since the EMG can be considered as a zero-mean stochastic process, amplitude appears as proportional to the standard deviation (STD) varying in time. Under this assumption, Mean Absolute Value (MAV) and Root Mean Square (RMS) were compared in [15] as maximum likelihood estimators of the EMG amplitude (and, consequently, EMG-muscle force relation). The previously mentioned reference reports experimental evidence giving a slightly better performance of MAV over RMS. MAV is defined as in Zecca *et al*. [16],

(1) $$MAV(k)=\frac{1}{k}\sum_{j=1}^{k}abs\left(emg(j)\right)$$

where *emg*(*j*) stands for the *j-th *sample from the beginning of the experiment and *k *is the current sample. This equation was modified to be applied in a recursive way, that is, more suitable for real-time control, that is,

(2) $$MAV(k)=\frac{k-1}{k}MAV(k-1)+\frac{1}{k}abs\left(emg(k)\right)$$

where *k *= 1,2,... corresponds to the sample time and *emg*(*k*) is the myoelectric signal in each sampled time.

**Figure 3:**
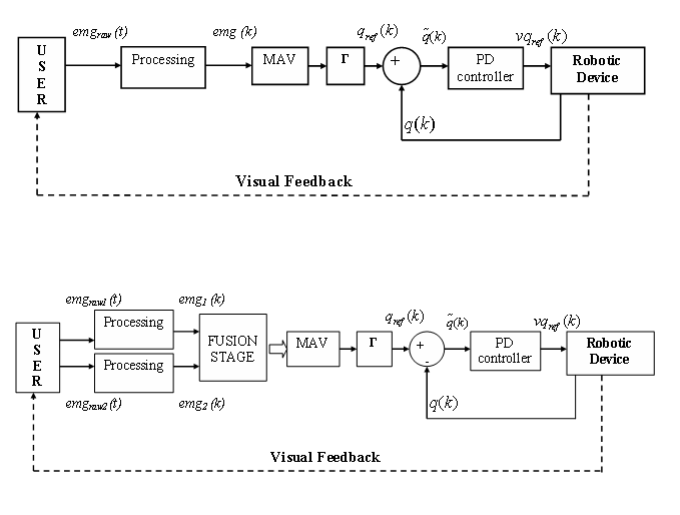
**Flowchart describing the proposed myoelectric control**. Top, the classical scheme for a single channel. The Processing stage correspond to Fig.2 and Table 1, MAV block extracts an estimate of muscular force, which is transformed to joint coordinates trough Γ. Bottom, the proposed redundant arrangement. Two channels or acquisition are fused, providing a single input signal to the MAV estimator.

The muscle contraction amplitude is estimated through MAV, which, in turn, is transformed to angular reference (*q*_*ref*_) for the controllers of the robot joint with a gain adaptation, i.e.: *q*_*ref *_= Γ*MAV *[Fig. 3]. A set of optical encoders provide the joint's position (*q*) for the error signal ($\tilde{q}(k)$) calculation. The entire system was implemented with two personal computers connected via the TCP/IP protocol. The first computer acquires the EMG, carries out the processing and calculates the coordinates for the robot joints. The second computer commands the robot with a Proportional Derivative (PD) control law [17], where $\tilde{q}(k)$ is the error signal and the output *vq*_*ref*_(*k*) stands for the manipulator's joint velocity reference. The robot reaches a position in the *x-y *plane according to the reference of *q*_*ref*_(*k*). The information of the joint positions and the direct kinematic equations was used to simulate the trajectory on the *x-y *plane and test its behavior. In addition, the volunteer performs a virtual training with a 3D simulator, seeing all robots' movements on a computer screen. The data are transmitted at a frequency of 1 KHz to prevent any incompatibility with the acquisition stage. Figure 3 displays an overview of the entire system.

### Methodology

Previous informed consent, biceps and triceps EMG signals were recorded during voluntary contractions in 4 normally limbed subjects (3 male, one female, 25 ± 3 years old) and one above elbow amputee (male, 24 years old).

After a period of rest, during which the background noise was recorded, volunteers were instructed to perform a 1s MVC with each muscle to be used in the normalization stage. During the test, the subject was instructed to use this pair of agonist-antagonist muscles to command the manipulator and displace its end effector to the right and left on the workspace. Both statics and dynamics contractions were tested while the type of contraction was chosen by the user.

The decision criterion for planning the trajectory is the sign obtained from the difference between MAV signals of the biceps and the triceps, that is, *sign*(*emg*_*channel*1_(*k*) - *emg*_*channel*2_(*k*)). The following (arbitrary) criterion was adopted: a biceps contraction causes a rightward displacement of the robotic arm, and the triceps contraction a displacement to the left.

### Algorithms

Two algorithms were proposed: Variance Weighted Average (VWA) and Decentralized Kalman Filter (DKF). Since the EMG signal recorded during voluntary dynamic contractions can be considered as a band-limited zero-mean Gaussian process, modulated by muscle activity and corrupted by a Gaussian additive white noise [18], its instantaneous changes of variance provide an indicator of muscle activity as well as the presence of fault-induced noise. For this reason, the variance was chosen as weighting function.

In what follows, *emg*_*i*_(*k*) denotes the value of the EMG signal in channel *i *at the time of step *k*. With this value, the recursive computation of the instantaneous temporal mean (average) signal ${\bar{emg}}_{i}(k)$(*k*) and the instantaneous variance $\sigma_{emg_{i}}^{2}(k)$(*k*) was calculated for each sample time *k*, that is,

(3) $$\bar{emg_{i}}(k)=\bar{emg_{i}}(k-1)+\frac{1}{k}\left(emg_{i}(k)-\bar{emg_{i}}(k-1)\right)$$

(4) $$\sigma_{emg_{i}}^{2}(k)=\sigma_{emg_{i}}^{2}(k-1)+\frac{1}{k}\left({\left[emg_{i}(k)-\bar{emg_{i}}(k)\right]}^{2}-\sigma_{emg_{i}}^{2}(k-1)\right)$$

#### VWA

In the first algorithm, a modified average was used, i.e.,

(5) *VWA(k) *= *w*_1_*(k)emg*_1_*(k)*+*w*_2_*(k)emg*_2_*(k)*

where *w*_1_(*k*) and *w*_2_(*k*)stand for the signal weights, respectively, and represent the normalized coefficients, which are variable with the variance of the signals $\sigma_{emg_{1}}^{2}(k)$(*k*) and $\sigma_{emg_{2}}^{2}(k)$(*k*) in the time step *k*

(6) $$w_{1}(k)=\frac{\sigma_{emg_{2}}^{2}(k)}{\sigma_{emg_{1}}^{2}(k)+\sigma_{emg_{2}}^{2}(k)},w_{2}(k)=\frac{\sigma_{emg_{1}}^{2}(k)}{\sigma_{emg_{1}}^{2}(k)+\sigma_{emg_{2}}^{2}(k)}$$

where both coefficients *w*_1_(*k*) and *w*_2_(*k*) satisfy the following conditions,

**0 **= *w*_1_*(k)*,*w*_2_*(k) *= **1**

*w*_1_*(k) *+ *w*_2_*(k) *= **1**

To clarify this concept, we give an analytical example, i.e.,

$$\begin{array}{l} \text{If }\sigma_{emg_{2}}^{2}(k)\gg \sigma_{emg_{1}}^{2}(k)\text{ then }VWA(k)≅emg_{1}\\ \text{If }\sigma_{emg_{2}}^{2}(k)\approx \sigma_{emg_{1}}^{2}(k)\text{ then }VWA(k)≅\frac{emg_{1}+emg_{2}}{2} \end{array}$$

#### DKF

Stochastic estimation tools such as the Kalman filter can be used to combine or fuse information from different media or sensors for hybrid systems. The Decentralized Kalman Filter (DKF) generates the overall signal estimate by minimizing the variances [6]. The DKF can be considered an algebraic equivalent of the Centralized Kalman Filter (CKF). Theoretically, there is no performance loss in the decentralized system, it delivers the same results as the CKF, but the benefits of the DKF are the modular concept that allows to add more sensors to the system, as needed, and an easier parallel implementation [18].

**Figure 4:**
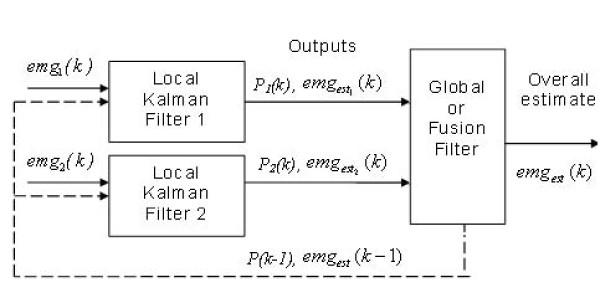
**Outline of a Decentralized Kalman Filter**. Each local filter produce estimates $emg_{est_{1}}(k)$(*k*) and $emg_{est_{2}}(k)$(*k*) based on the information available from *emg*_1_(*k*) and *emg*_2_(*k*) using the standard Kalman Filter equations. The Global Filter block fuses these estimates together to form the overall state estimate *emg*_*est*_(*k*).

Figure 4 summarizes the concept of a DKF, where the local filter outputs converge to the overall fusion filter via the respective variance and the estimated local outputs. In fact, as mentioned above, many local filters can be added as needed, and always the data are fused at the final filter.

As in the previous algorithm, the instantaneous variance is the decision parameter. Therefore, instantaneous mean and variance must be recursively computed with equations (3) and (4), thereafter; these values are inserted in the local filter (7). Finally, the vectors are fused in the overall filter (8), according to the procedure described by Soria et al. [7],

(7) $$\begin{array}{c} P_{i}^{-1}(k)=P_{i}^{-1}(k-1)+{\left(\sigma_{i}^{2}\right)}^{-1}\\ emg_{est_{i}}(k)=P_{i}(k)\left(P_{i}^{-1}(k-1)emg_{est_{i}}(k-1)+{\left(\sigma_{i}^{2}\right)}^{-1}emg_{i}(k)\right) \end{array}$$

(8) $$\begin{array}{c} P^{-1}(k)=P^{-1}(k-1)+\sum_{i=1}^{n}P_{i}^{-1}(k)-P_{i}^{-1}(k-1)\\ emg_{est}(k)=P(k)\left(P^{-1}(k-1)emg_{est}(k-1)+\sum_{i=1}^{n}P_{i}^{-1}(k)emg_{est_{i}}(k)-P_{i}^{-1}(k-1)emg_{est_{i}}(k-1)\right) \end{array}$$

where *i *represents the local filter, *n *is the number of local filters,*P*_*i *_stands for the local variance, $emg_{est_{i}}(k)$(*k*) is the filtered (estimated) signal, *P *represents the global variance, and *emg*_*est*_(*k*) describes the global estimated vector.

## Results

### Analysis of both algorithms in the presence of failures

**Figure 5:**
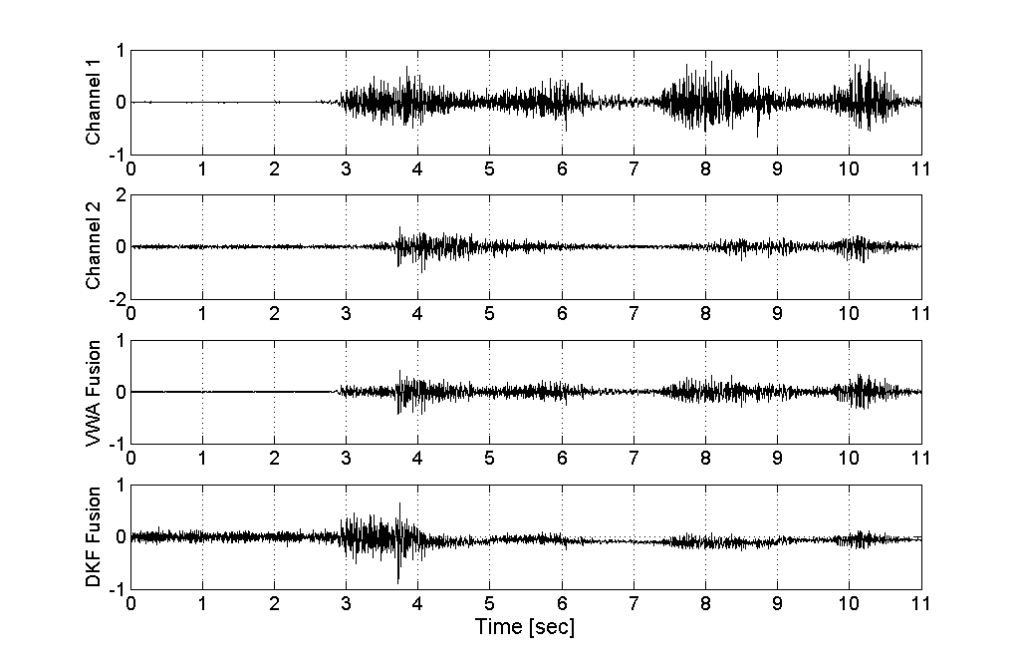
**Normalized EMG signals from biceps and results of the fusion for an arrangement of two bipolar electrodes, in normal conditions**.

**Figure 6:**
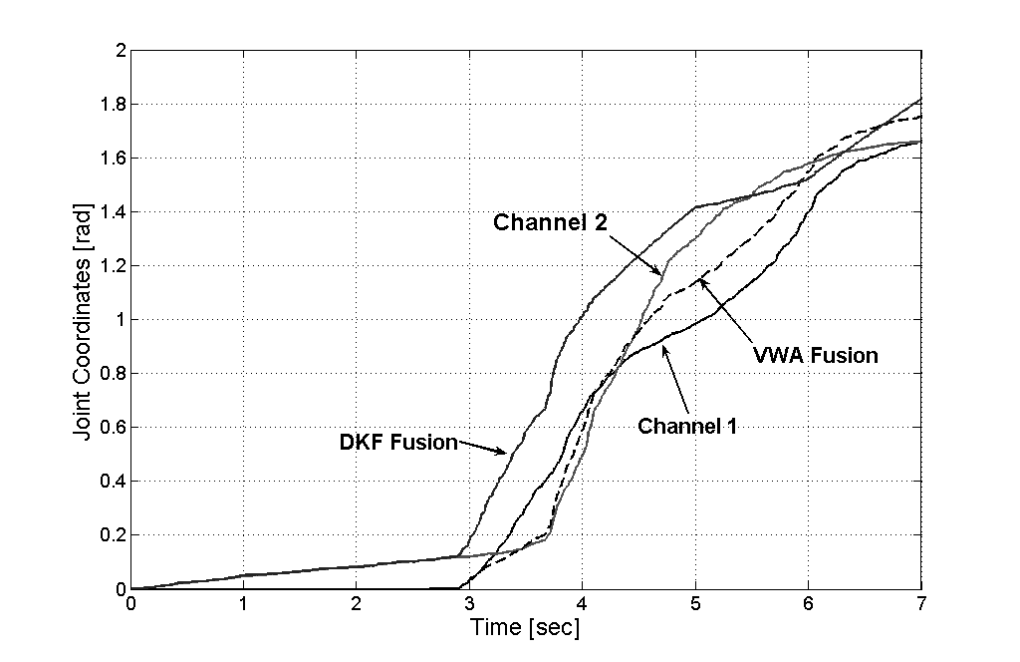
**Joint coordinates movements as functions of time obtained from the signals of Fig. 5 and their fusion**.

Several trials, all well-accepted by the volunteers, were performed with this procedure. A representative experiment carried out with one array of acquisition channels (from the biceps) clearly shows the fusion results (Figs. 5 and 6). Even when the two signals were recorded from the same muscle, their shapes and amplitudes were not identical, because of the different sensing sites. However, the signal used to command the robot is the result of their fusion, in such a way that differences were smoothed by the averaging effect of the filter. The operator visual feedback of the manipulator minimizes any difference between the signals from each pair and the global fusion (see Fig. 3 and Fig. 6).

Once both algorithms were adjusted, they were experimentally tested under several conditions, as for example, disconnecting and reconnecting one electrode in an alternating manner (a temporary electrode disconnection leads to saturation of the amplification stage). The EMG without perturbations (called noiseless channel) was continuously recorded as reference while the noisy signal was introduced into the robot mathematical model to compute the trajectory with failures. Both graphs were thereafter analyzed for evaluation purposes.

**Figure 7:**
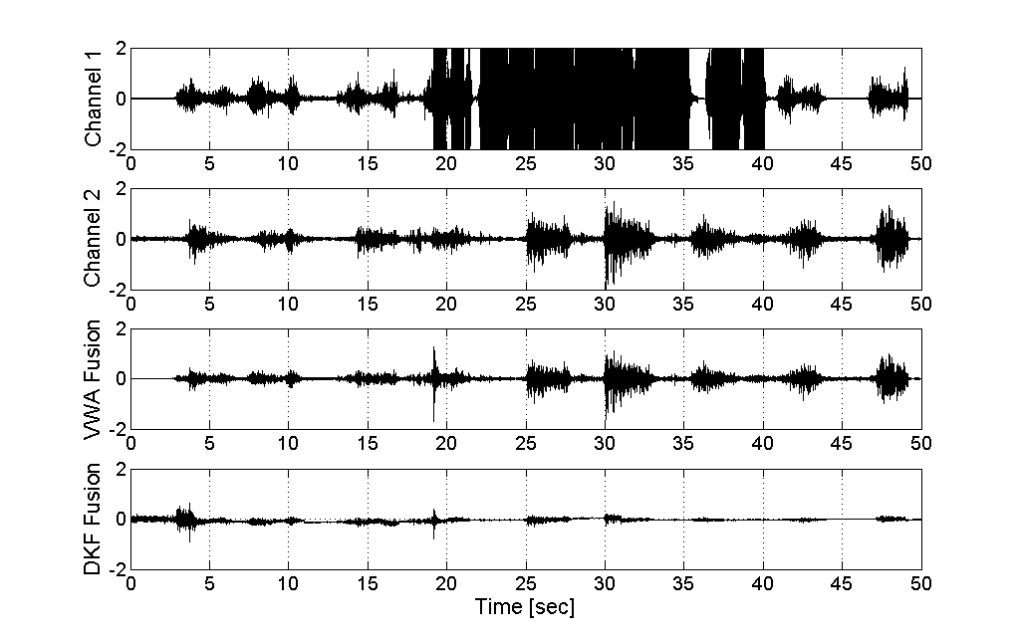
**Fusion of EMG's**. Top: Channel 1, normalized biceps EMG saturated during disconnections. Second: Channel 2, normalized noiseless signal. Third: Channel 3, Fusion signal from VWA. Bottom: Channel 4, Fusion signal from DKF. The DKF Fusion presents an important attenuation.

**Figure 8:**
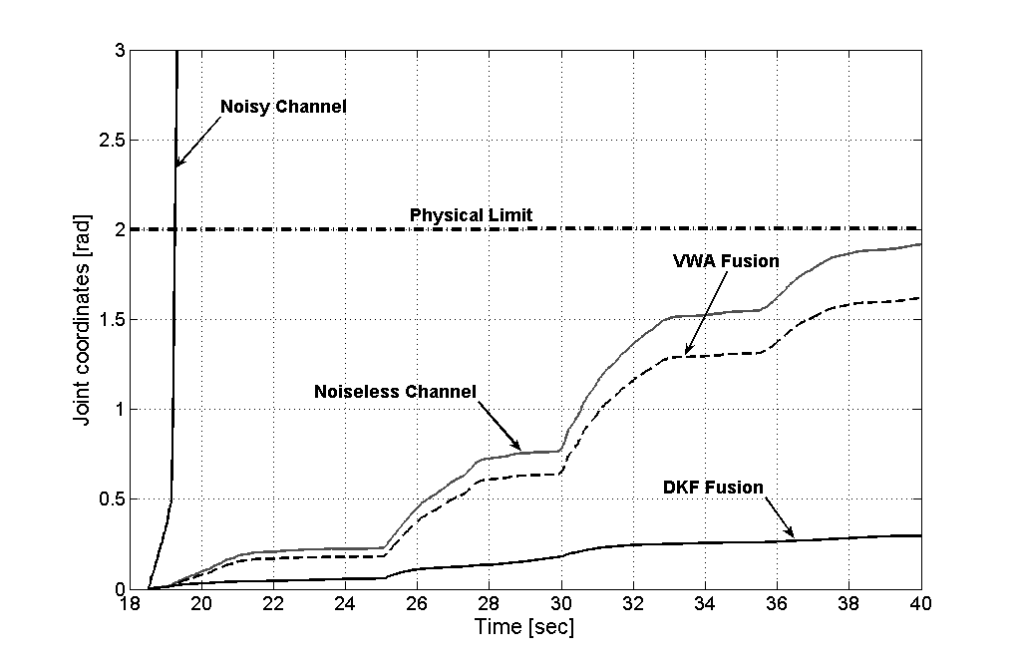
**Joint coordinates for the EMG fusion from the moment of electrode disconnection**. The dotted gray line marks the physical limit of the robot. Beyond this limit, the safety mechanisms are activated.

The resulting EMG after applying both algorithms is presented in Figure 7. In Channel 1, the electrode was disconnected and reconnected several times leading to saturation, while in the second channel the noiseless signal remained with no changes. The third and fourth channels show the results of VWA and DKF fusions, respectively. The results are not identical, but the transformation to robot control action is similar, as shown in Figure 8.

Figure 8 shows the results of fusion transformed into joint coordinates from the electrode disconnection instant. Both algorithms produced signal attenuation caused by the averaging effect of VWA and the inherent filtering of DKF fusion. However, the inclusion of an amplification stage could greatly mitigate this effect, but the result in stationary state, without failures, would be modified (see Fig. 6).

Even when the transformation of this signal to joint coordinates is not exact, the failure is ignored by the control system, and the joint reference signal never exceeded the workspace limits. Under these circumstances, the electrode disconnection can be detected by the operator and therefore corrected.

**Figure 9:**
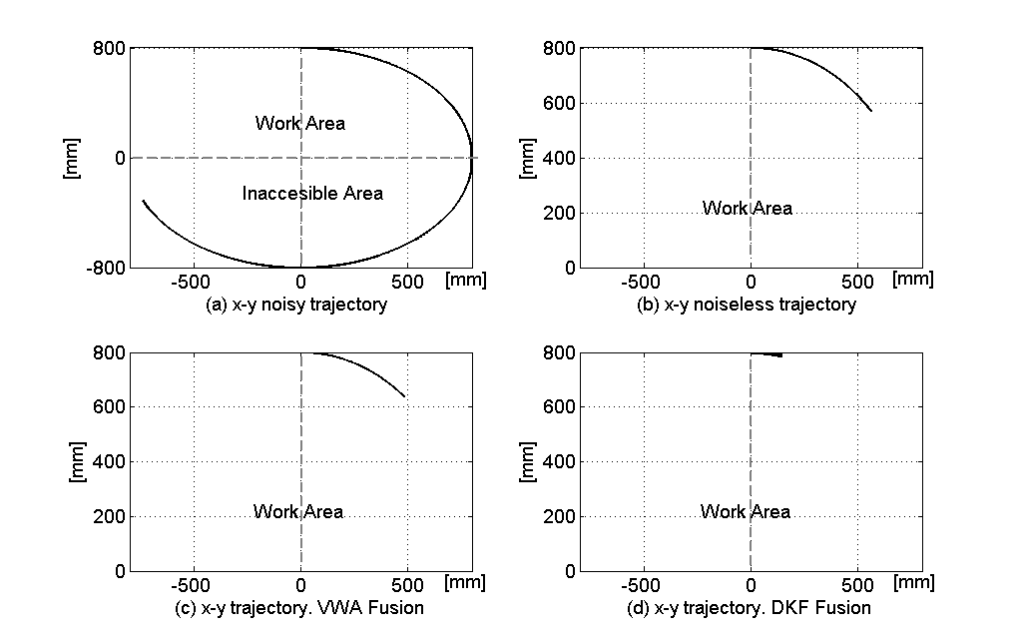
**Planar trajectories for the signals of Fig. 7 and for results of Fig. 8**.

Figure 9 displays the x-y trajectories for noisy (9-a), noiseless (9-b), VWA and DFK fused signals (9-c and 9-d). The path generated by the contaminated signal (first channel, Fig.7) exceeded widely the robotic arm range; nevertheless, the commands coming from either VWA or DKF fusion were kept inside the workspace limits, in accordance with the joint coordinates of Figure 6.

**Figure 10:**
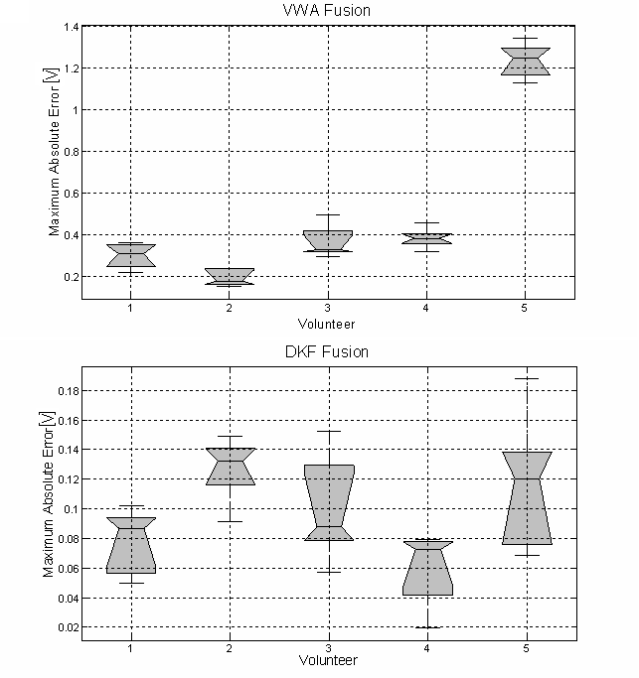
**Errors in the computation of joint coordinates in two consecutive and similar contractions, the second under noisy conditions**. The values given in these boxplot diagrams (from bottom to top: 5th, 25th, 50th, 75th, or 95th percentiles) are the median values across five repetitions in each one of the five volunteers. Top: Statistical results of VWA Fusion, Bottom: Statistical results of DKF Fusion. (See also Results Section-A).

In order to demonstrate the results in all volunteers, they were instructed to perform two consecutive and similar contractions while the electrode was removed in channel 2 during the second contraction. This procedure was repeated five times with each volunteer. Under the assumption that both contractions generate similar (or quasi identical) joint coordinates, the result of fusion was compared through the maximum absolute error between the first coordinate value (noiseless contraction) and the second, with electrode disconnection. The statistical analysis showed more dispersion in DKF than in VWA, even when the error in both techniques was not significant (Fig. 10).

### Performance analysis under different noise conditions

The two fusion methods were evaluated by applying real EMG signals, which had been separately corrupted with a range of white noise and 50-Hz interference. In the first case, we applied a stationary white noise, which has a standard deviation depending on the noise level, while in the second case, instead, we used different levels of power line interference (0 to 10 V, in steps of 1 V). The power line interference was simulated by a sinusoidal signal of 50 Hz with adjustable amplitude. For both algorithms, the absolute error maximum of the generated noisy signals was computed with Equation 9. Results are summarized in Table 2.

**Table 2:** Maximum absolute error (Eq.9) for both algorithms, when the EMG signals were corrupted by Gaussian white noise, and by power line interference.

Noise Level	Gaussian white Noise		50 Hz Interference	
	
[mV]	VWA	DKF	VWA	DKF
0,1	0,173	0,973	0,077	0,605
0,2	0,243	0,711	0,090	0,683
0,3	0,282	1,096	0,096	0,745
0,4	0,418	0,692	0,096	0,783
0,5	0,401	1,146	0,094	0,806
0,6	0,709	0,934	0,098	0,819
0,7	0,674	1,038	0,106	0,827
0,8	1,106	1,038	0,121	0,832
0,9	0,540	1,021	0,135	0,835
1,0	0,781	0,787	0,149	0,838

(9) max *abs error *= max(*abs*(*emg*(*k*) - *emg*_*est*_(*k*))

The procedure described in Section III-A was repeated in all volunteers to compute the average of the absolute error (i.e. infinity norm) for both methods. The maximum absolute error for DKF Fusion was 1.6285 V (STD 0.13) and for VWA was 0.3635 V (STD 0.47), in the group of healthy subjects. The same calculation was made for the amputee; in this case the error for DKF Fusion was 1.978 V and for VWA was 0.4275 V. There are no significant differences, thus the performance is similar in both groups.

## Discussion

The literature does not seem to be abundant in the use of fusion for EMG signals. We could find the report by Silva et al. [19] applying data fusion of mechanomyography (MMG) signals for prosthesis control. These authors concluded that a multisensor data fusion technique is used as strategy for the generation of binary control signals for an electrically powered prosthesis, none the less, this paper is somewhat unclear in its results and is not well related to the way we use it here.

When the benefits of each algorithm are analyzed, as in any robustness scheme, two aspects must be taken into account: noise sensitivity and computational cost. On one hand, VWA appears as more efficient due to its better sensitivity to noisy signals, but the DKF algorithm is recommended in fusion where the signals come from sensors whose nature is different, like electrodes for electromyography, piezoelectric contact for mechanomyography, accelerometers for acceleromyography (AMG), and condenser microphones for phonomyography (PMG). On the other hand, the computational cost for both algorithms is the same, therefore, this is not a decision factor, and the choice would depend on the expected perturbations and the possibility of incorporating new sensors.

The use of redundant potentials is ultimately limited by the practical possibility of sensing with two or more electrodes on the same muscle group. However, this is not always feasible in amputees because of the shape and space availability on the stump area for attachment of surface electrodes and the problem of adhesion.

The two proposed fusion algorithms, VWA and DKF, have demonstrated an efficient performance. Despite the fact that both algorithms have shown different responses to noise, the manipulator never moved beyond its safety range. Moreover, the true system trajectories followed closely the ideal trajectories generated with the robot mathematical model. This outline could be applied to myoelectric control of prosthesis, or assistive manipulators in order to assure the functionality under electrode faults and noisy environments.

## Conclusion

Two data fusion algorithms of EMG signals are proposed in this paper with the aim of improving the fault robustness and safety characteristics of a myoelectric controlled manipulator system. The major advantages for this scheme are: the continuous operation of the manipulator, even in case of electrode disconnection, and the modularity that offers the possibility to include different number and types of sensors. The main contribution of the work proposed here can be centered around two main issues. First, the improvement of the robustness preventing potential risks for the operator and the environment in case of failure, tested under real and simulated noisy conditions. Second, the fact that two simple data fusion algorithms based on the instantaneous variance analysis and without computational cost were applied to EMG.

The two algorithms used demonstrated an acceptable performance.

## Competing interests

The authors declare that they have no competing interests.

## Authors' contributions

NML conceived, designed and implemented the algorithms, adquired the data and drafted the manuscript. FDS supervised the project, contributed to the design of the algorithms. CMS programmed the control of the robot and the DSP, participated and contributed to the discussion and interpretation of the results. MEV supervised the project and the research group, and drafted the manuscript. All authors read and approved the final manuscript.
